# Safety and feasibility of early drinking water after general anesthesia recovery in patients undergoing daytime surgery

**DOI:** 10.1186/s12871-024-02615-5

**Published:** 2024-07-10

**Authors:** Yixing Lu, Siyan Liu, Shunzhong Jing, Xuefeng Zhao, Jiamei Liang, Xiaoqiang Sun, Yunan Lin

**Affiliations:** 1 Department of Anesthesiology, Maternal and Child Health Hospital of Guangxi Zhuang Autonomous Region, No. 225 Xinyang Road, Nanning, 530003 China; 2 Department of Anesthesiology, the First Affiliated Hospital of Guangxi Medical University, No. 6 Shuangyong Road, Nanning, 530021 China; 3 Department of Anesthesiology, Reproductive Hospital of Guangxi Zhuang Autonomous Region, Nanning, China; 4 Department of Anesthesiology, the First People’s Hospital of Yulin, Yulin, China

**Keywords:** General anesthesia, Early drinking water, Pre-drinking water assessment, Nausea and vomiting, Antral motility index

## Abstract

**Background:**

Patients who are recovering from general anesthesia commonly exhibit symptoms such as dry lips, throat irritation, and thirst, prompting a desire to drink water in the post-anesthesia care unit (PACU). In this study, we aimed to evaluate the therapeutic effects and any potential complications of administering varying quantities of water to such patients. The primary objectives are to assess the safety and feasibility of early water intake after general anesthesia, specifically in the context of daytime surgery.

**Methods:**

A total of 200 nongastrointestinal patients who underwent outpatient surgery were randomly assigned to four groups: Group A (drinking < 1 ml/kg), Group B (drinking 1–2 ml/kg), Group C (drinking > 2 ml/kg), and Group D (no water intake). We monitored changes in the assessment parameters before and after water consumption, as well as the incidence of post-drinking nausea and vomiting, and compared these outcomes among the four groups.

**Results:**

Water intake led to a significant reduction in thirst, oropharyngeal discomfort, and pain scores and a notable increase in the gastric antrum motility index (MI), exhibiting statistical significance compared to the values before drinking (*p* < 0.05). Remarkably, higher water consumption correlated with enhanced gastrointestinal peristalsis. There was a significant difference in the antral MI among groups B, C, and A (*p* < 0.05). The occurrence of nausea and vomiting did not significantly differ among groups A, B, C, and D (*p* > 0.05). Early water consumption enhanced patient satisfaction with medical care, significantly varying from Group D (*p* < 0.05).

**Conclusion:**

Non-gastrointestinal surgical patients who passed pre-drinking water assessments post GA(general anesthesia)recovery could safely ingest moderate amounts of water in the PACU. Early water intake is both safe and feasible, effectively fostering swift postoperative recovery.

## Introduction

According to the standard requirements of daily surgical anesthesia management, patients who have undergone general anesthesia are conventionally subjected to preoperative fasting and prohibited from drinking water. Patients who have not been drinking water for a long time have lost body fluids. Therefore, most patients have dry lips and parched mucous membranes upon awakening from general anesthesia. After surgery, patients experience considerable thirst, throat discomfort, emotional instability, and other similar discomfort, which significantly heightens their desire for water consumption.

Postoperative dietary management is one of the core components of enhanced recovery after surgery (ERAS) management [1]. Clinical research has provided evidence on the advantages and safety of reintroducing oral fluids after patients undergo general anesthesia. The early postoperative resumption of oral food and fluid intake can facilitate the recovery of intestinal motor function and accelerate the overall postoperative recovery of the body [2].

However, there is a notable scarcity of clinical studies focusing on early postoperative water intake. Notable discrepancies exist in terms of the timing of water consumption, the quantity of water intake, and the method of drinking water [3, 4]. Existing evidence suggests that patients in the early stages of recovery from general anesthesia can consume an appropriate amount of water. Both children and adults have reported the ability to drink water shortly after recovery from general anesthesia [5, 6]. Nevertheless, there is a considerable dearth of substantial research addressing the quantity and timing of water consumption after surgery. The feasibility of water consumption upon request and the consequences of excessive water intake remain uncertain. There is a clear need for multicenter, large-sample, and randomized controlled studies. Thus, we conducted a prospective randomized controlled study to explore the safety and feasibility of early PACU water consumption for patients recovering from general anesthesia and to provide practical insights for accelerating the clinical progress of rehabilitation surgery.

## Materials and methods

This forward-looking, randomized, controlled study received ethical approval from the Scientific Research Project Ethics Committee of the First Affiliated Hospital of Guangxi Medical University (No. 2022-KY-(087), 26 August 2022). It was registered at chictr.org.cn (6 September 2022; identification number: ChiCTR2200063418) before the start of the trial and patient enrollment. Written informed consent was obtained from all patients.

### Participants and inclusion/exclusion criteria

**Fig. 1:**
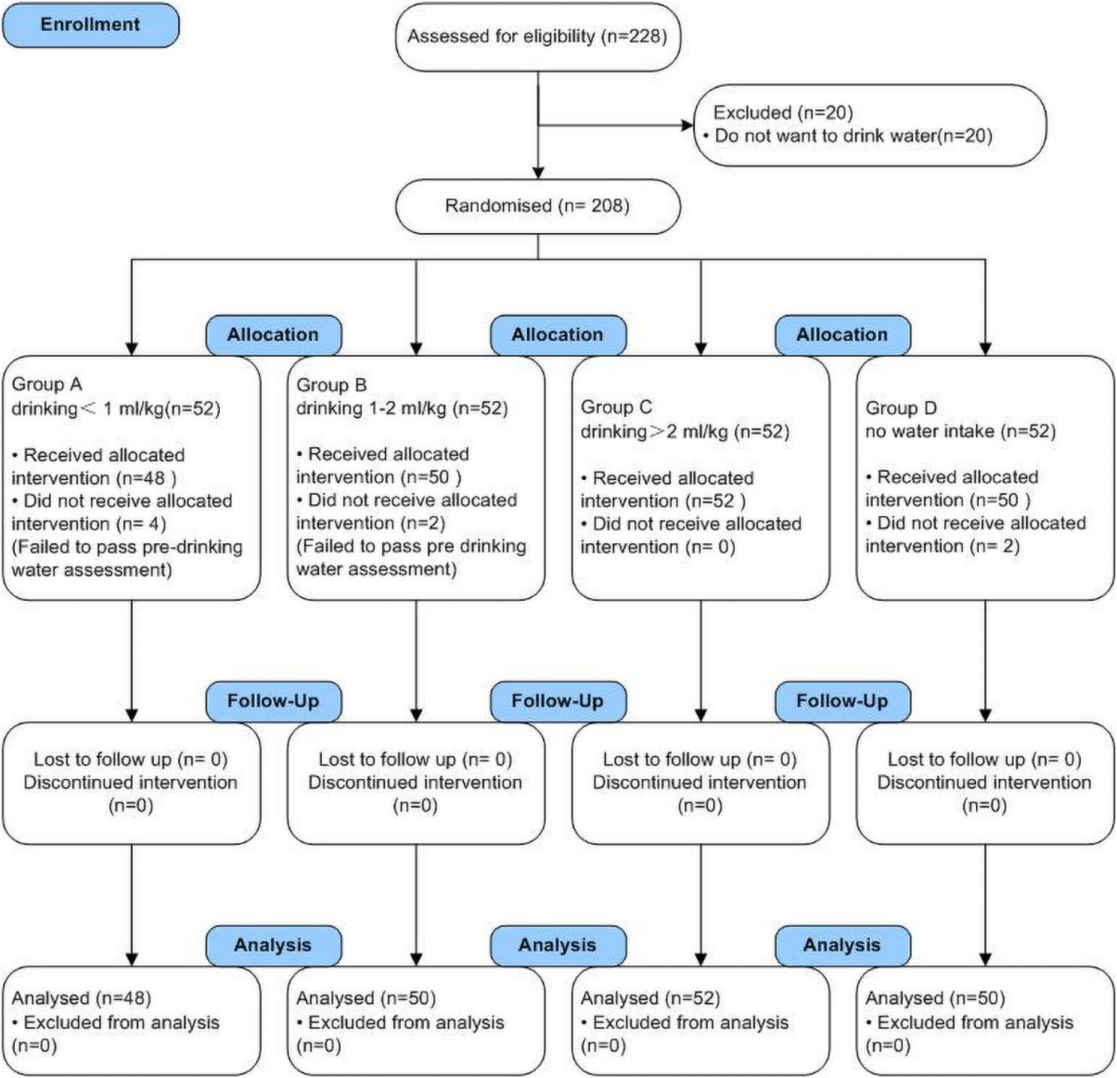
CONSORT flowchart of the study

A total of 228 patients of either sex, aged ≥18 years, with ASA I-II who underwent non-gastrointestinal surgery were included in the study from September 1, 2022, to May 1, 2023. The exclusion criteria included challenging intubation, anesthetic issues, and refusal to participate in the study for any reason. The study adheres to the CONSORT guidelines for randomised controlled clinical trials, the CONSORT flowchart is reported in Fig. 1.

### Sample size calculation

This study is a randomized controlled trial. The intervention group receives a specific treatment with varying water intake, while the control group receives no treatment. The main observed indicator is the vomiting rate of the subjects. Based on the literature review, an expected vomiting rate of 3.7% is anticipated in the intervention group, while the prevalence in the control group is 23.6%. A bilateral α = 0.05 is established with an 80% confidence level. The sample size is determined using the prescribed sample size calculation formula.

$$n=\frac{{2\bar {p}\bar {q}{{\left( {{Z_\alpha }+{Z_\beta }} \right)}^2}}}{{{{\left( {{p_1} - {p_2}} \right)}^2}}}$$

The calculation results indicate that there are 47 cases with a requirement of 47 subjects in each of the intervention and control groups under 1:1 randomization grouping. Factoring in a 15% loss of follow-up and refusal to visit, the minimum total number of subjects needed in both groups is 55, totaling at least 221 subjects.

### Randomization and intervention

Before anesthesia, researchers informed eligible patients about the possibility of consuming moderate amounts of water while awake, detailing the specific protocols and observations regarding water intake. However, the patients were not informed about the quantity of water they could consume. Following surgery, patients were moved to the PACU for anesthesia recovery. Researchers then reassessed the awake patients, inquiring about their desire to drink water. Patients who fulfilled the water intake criteria and expressed a desire to drink water were randomly assigned to different groups. Combining the recommendations of relevant literature on water intake, block randomization was utilized to create a random allocation sequence for each block, with a size of four participants per block. The random sequence within each block was then employed to assign participants consecutively into four groups: Group A receiving less than 1 ml/kg (*n* = 48); Group B, receiving 1–2 ml/kg (*n* = 50); Group C, receiving more than 2 ml/kg (*n* = 52); and Group D, receiving no water (*n* = 50).

### Standard assessment protocol

The assessment process advanced through sequential stages:

- First, the assessment involved examining the patient’s level of consciousness, respiratory status, ability to protect the airway (coughing and swallowing), and movements of the head and limbs [7, 8].
- Subsequently, we assessed the patients’ circulation, SPO_2_ (pulse oxygen saturation) levels, occurrence of postoperative nausea and vomiting, and any signs of bleeding or discharge at the surgical site post-general anesthesia recovery [8, 9].
- Ultimately, following successful recovery, the 3-ounce water swallow test [10] was performed to gauge the patient’s drinking and swallowing capacities after any swallowing challenges were resolved.

### Data collection

**Fig. 2:**
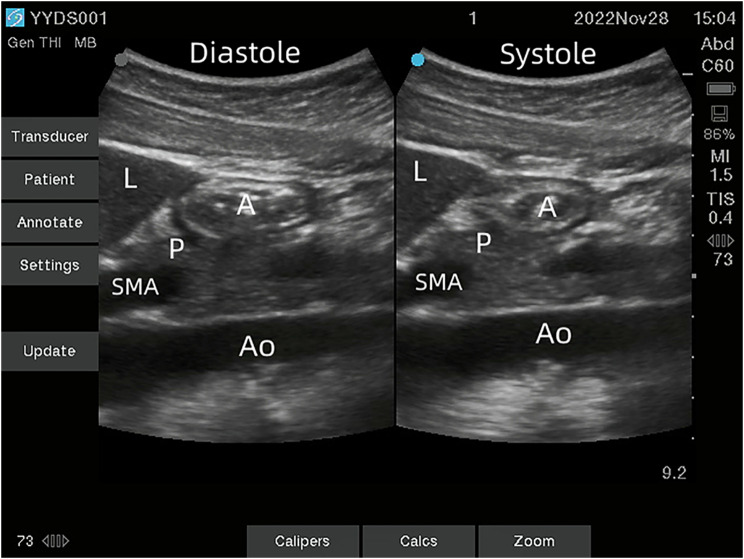
Diastolic and systolic ultrasound images of the gastric antrum after drinking. L: liver; A: gastric antrum; P: pancreas; SMA: superior mesenteric artery; Ao: abdominal aorta

In this study, the data collectors were not blinded to the study protocol. The primary outcome was the incidence of nausea and vomiting associated with drinking different amounts of water. The secondary outcome parameters were patient experiences before and after drinking, including facial expression, thirst, oropharyngeal discomfort, and postoperative pain. Ratings were depicted on a scale from 0 to 10, where higher scores denoted more severe conditions. A modified B-ultrasound technique [11] was employed to measure the antral MI for scanning (Fig. 2).

### Statistical analysis

Categorical data are presented as frequency percentages (n %) and were analyzed through the chi-square (χ^2^) test. Normally distributed continuous data are expressed as the mean ± standard deviation (± *s*). Intragroup comparisons were performed using paired sample t tests, whereas multigroup comparisons were performed with nonparametric tests. For nonnormally distributed continuous data, the data are presented as the median and interquartile range, with *p* < 0.05 indicating statistical significance. Pearson Correlation Coefficient is employed to evaluate the intensity and direction of the linear correlation between two numerical variables.

## Results

A comparative analysis of the four study groups is presented in Table 1. There were no notable differences in the initial characteristics of the patients, duration of abstinence from alcohol, anesthesia duration, intraoperative fluid volume, first oral hydration time, or surgical procedure type (*p* > 0.05). The bed was elevated during water consumption. Individual patient needs varied, leading to variations among groups (*p* < 0.05).

**Table 1:** Patient characteristics and anesthesia management

	Group A	Group B	Group C	Group D	*P* value
	(*n* = 48)	(*n* = 50)	(*n* = 52)	(*n* = 50)	
Male/female	22/26	29/21	32/20	27/23	0.437
Age (years)	34.73 ± 9.97	36.48 ± 11.99	34.29 ± 10.51	35.32 ± 9.57	0.772
Height (cm)	164.67 ± 8.58	165.62 ± 8.55	167.06 ± 9.39	166.08 ± 8.14	0.563
Weight (kg)	60.51 ± 12.35	64.22 ± 12.59	63.90 ± 14.19	63.96 ± 12.44	0.396
BMI (kg/m^2^)	22.11 ± 2.81	23.33 ± 3.75	22.69 ± 3.52	23.05 ± 3.23	0.511
Prohibition duration (h)	11.64 ± 3.53	11.58 ± 3.78	11.45 ± 2.51	11.63 ± 1.58	0.972
Anesthesia duration (min)	125.63 ± 38.54	124.84 ± 41.25	128.04 ± 44.99	128.68 ± 26.00	0.646
Intraoperative infusion	315.21 ± 115.21	328.00 ± 118.30	325.00 ± 132.66	316.50 ± 85.70	0.956
Volume (ml)					
First oral hydration time (min)	32.6 ± 1.3	32.7 ± 1.5	29.0 ± 1.0	30.2 ± 1.2	0.063
Head of bed elevation (°)	16.2 ± 1.1	19.6 ± 0.9	21.1 ± 1.1		0.009*
Surgical type					
Arthroscopic surgery n(%)	12 (25.0)	15 (30.0)	18 (34.6)	15 (30.0)	0.879
Thyroid surgery n(%)	23 (47.9)	16 (32.0)	11 (21.2)	20 (40.0)	
Laparoscopic surgery n(%)	4 (8.3)	6 (12.0)	6 (11.5)	4 (8.0)	
Internal Fixation Removal n(%)	5 (10.4)	4 (8.0)	6 (11.5)	4 (8.0)	
Superficial lumpectomy n(%)	0 (0.0)	4 (8.0)	3 (5.8)	3 (6.0)	
Others n(%)	4 (8.3)	5 (10.0)	8 (15.4)	4 (8.0)	

**Fig. 3:**
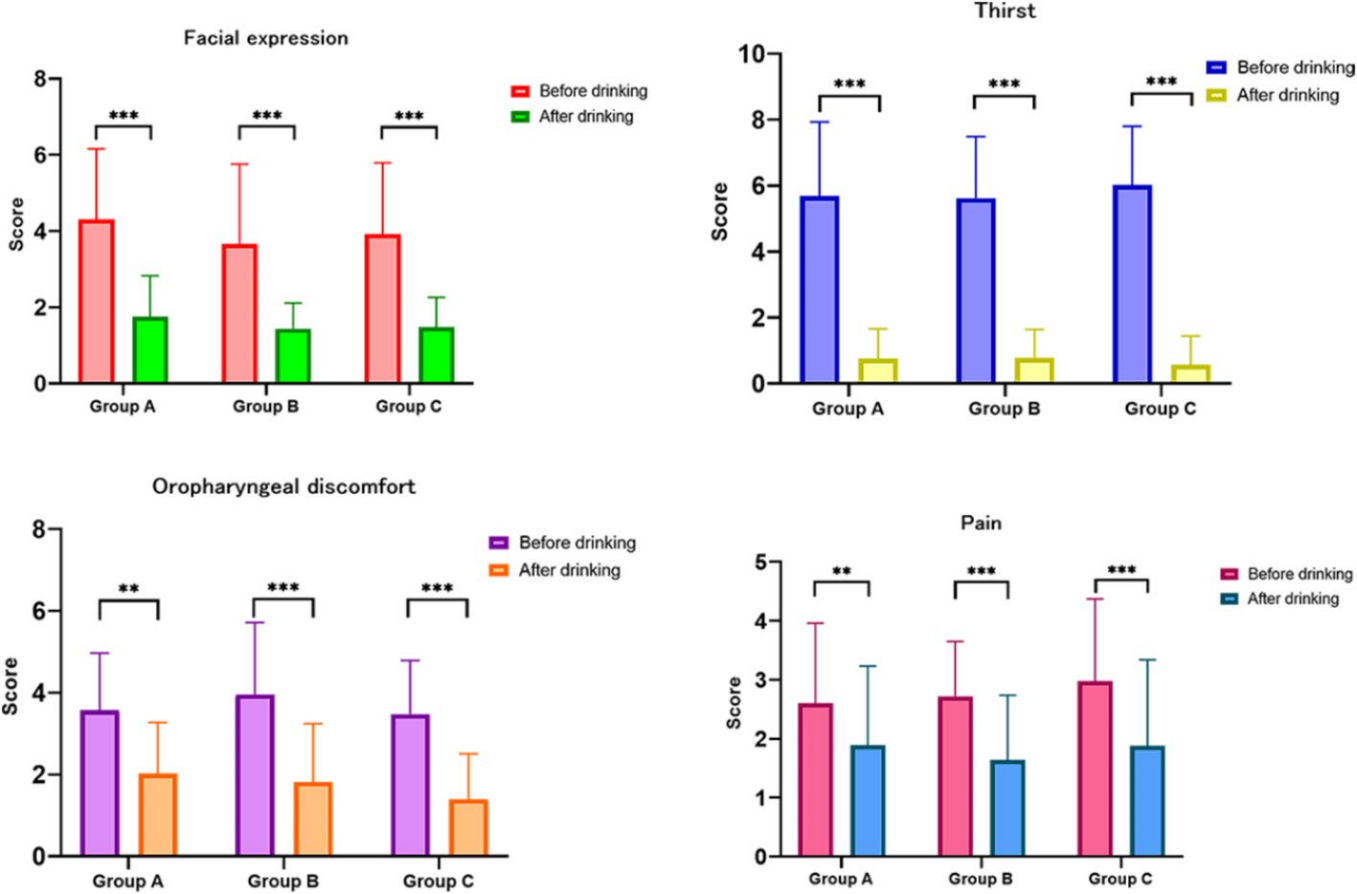
Evaluation indices before and after drinking

**Fig. 4:**
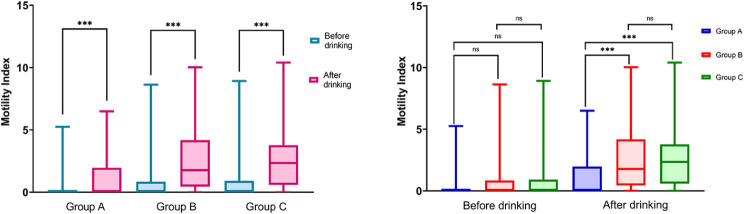
Antral MI before and after drinking

The occurrence of nausea and vomiting was not significantly different among the four groups of patients (*p* = 0.289; Table 2). Although there was no clear link between individual treatment indices and fluid intake, a notable positive relationship was observed between the antral MI and water consumption (Table 3). The scores of each index were lower after drinking water than before, for facial expressions (1 [IQR 1–2] vs. 4 [3–5]; *p* < 0.001), thirst (0 [IQR 0–0] vs. 6 [5–8]; *p* < 0.001), oropharyngeal discomfort (2 [IQR 1–2] vs. 3 [3–5]; *p* < 0.001), and pain (2 [IQR 1–2] vs. 3 [2, 3]; *p* < 0.001; Fig. 3). Moreover, the antral MI significantly improved after water consumption (1.61 [IQR 0-3.66] vs. 0 [0-0.05]; *p* < 0.001). Furthermore, the greater the amount of water consumed, the more pronounced the improvement in the antral MI (*p* < 0.001; Fig. 4).

**Table 2:** Incidence of adverse events after water consumption [n (%)]

Adverse event	Group A (*n* = 48)	Group B (*n* = 50)	Group C (*n* = 52)	Group D (*n* = 50)	*P* value
Immediate nausea and vomiting	0 (0.0)	0 (0.0)	0 (0.0)	0 (0.0)	1.000
Ward nausea and vomiting	1 (2.1)	0 (0.0)	0 (0.0)	2 (4.0)	0.289
Abnormal exhaust/defecation	0 (0.0)	1 (2.0)	0 (0.0)	0 (0.0)	0.392
Poor appetite	3 (6.3)	1 (2.0)	2 (3.8)	1 (2.0)	0.626

**Table 3:** The relationship between water consumption and different metrics

	Facial expressions	Thirst	Oropharyngeal discomfort	Pain	Antral MI
**Water intake**	-0.051	-0.076	-0.186*	0.097	0.283**
***P-Value***	0.534	0.354	0.023	0.237	< 0.001

*Correlation is significant at the 0.05 level. **Correlation is significant at the 0.01 level

## Discussion

Accelerated surgical rehabilitation recommends the oral intake of clear fluids up to ≤ 400 mL 2 h before surgery [12]. Clinicians prioritize safety and may not always adhere to this preoperative fluid intake recommendation. Our study revealed that the majority of patients refrained from drinking water from the evening before surgery until the following day. Some patients consumed 400 mL of clear fluid at 06:00 a.m. on the day of the operation. On average, patients included in the study abstained from drinking for approximately 11.57 ± 2.95 h.

The ERAS management concept recommends goal-oriented or restricted fluid infusion during surgery, which significantly reduces the total intraoperative fluid volume in patients [13, 14]. In our collected patients, the average duration of anesthesia was 126.82 ± 38.18 min, with an intraoperative infusion volume of 321.28 ± 113.71 mL. Despite these anesthesia management strategies, they may not fully meet the fluid needs of patients who have refrained from consuming water for a long time. As a result, patients in the day surgery center frequently present with dry throats and a strong desire to drink water upon entering the PACU after recovering from anesthesia.

In this study, the time elapsed from the recovery of general anesthesia to the commencement of water intake averaged 31.77 ± 11.44 min. Various studies have reported different recommendations regarding the timing of water intake. Mercan proposed that children could drink water 1 h after surgery [5], whereas Yin’s study reported a first water intake time of 0.29 ± 0.14 h [15], which was similar to our results.

Before drinking water, patients are required to undergo a safety assessment, which we do in conjunction with the Safety Protocol for Thirst Management (SPTM) [8] and the postanesthetic exit scoring system [16]. The assessments included good recovery of consciousness, recovery of respiratory protection mechanisms (coughing and swallowing), and absence of postoperative nausea and vomiting. The assessment of not only the pharyngeal reflexes and swallowing function was emphasized but also postoperative bleeding from the surgical site, potential postoperative airway risks, and secondary surgical risk assessment. This study was conducted to ensure patient safety and provides a valuable reference for future clinical applications.

Studies have reported concerns regarding complications such as vomiting and accidental inhalation following early water consumption. Most studies recommend restricting the water supply in the PACU. In this study, patients were divided into four groups based on the amount of water they consumed. There was no statistically significant difference in the incidence of nausea and vomiting between the groups (*p* = 0.289). Patients did not show a greater tendency for complications such as choking, coughing, nausea, or vomiting despite consuming excessive amounts of water. For instance, 19.23% (11/52) of patients in Group C drank more than 400 ml of water in a single sitting position, with the highest intake recorded at 750 ml. Remarkably, no instances of nausea or vomiting were reported in Group C due to increased water intake. Only one patient from the consumption group vomited after returning to the ward, resulting in an overall occurrence rate of 0.67%, which was significantly lower than that reported in previous studies [17, 18]. In contrast, Group D included two cases of coughing and vomiting in patients who drank water after returning to the ward. All three patients underwent goiter resection. The primary reason for vomiting was not poor gastrointestinal function recovery but rather throat stimulation associated with this type of surgery. After complete metabolism and clearance of narcotic analgesic drugs during surgery, patients often experience significant swallowing pain. This made them prone to coughing and, subsequently, vomiting when drinking too much or too quickly. By adjusting the drinking water speed and the volume of water consumed in a single instance or by utilizing oral analgesics, these patients can effectively reduce their discomfort and minimize the risk of choking-cough-induced vomiting. The patients included in the study did not experience severe vomiting or accidental inhalation during their hospitalization.

Proper positioning during drinking is crucial, as adjusting posture correctly can enhance patient comfort, alleviate tension, and effectively reduce the likelihood of nausea and vomiting. By aligning the head position with physiological norms, we can alleviate strain on soft tissues such as neck muscle ligaments. This decrease in muscle tension and neck pain also helps reduce vertebral artery compression and distortion, thus preventing cerebral ischemia, lowering intracranial pressure, and minimizing the chances of headaches, nausea, and vomiting [19]. Moreover, adjusting the head position displaces the upper abdominal organs outward, relaxes abdominal muscles, stimulates the gastrointestinal tract, and can dampen certain vagal impulses responsible for nausea and vomiting. Therefore, it is important to gradually modify the head elevation to ensure a comfortable drinking posture for each patient. In our research, the head of the bed was raised at a 19-degree angle. However, the degree of bed inclination for head elevation is influenced by the surgical area. For instance, in regard to wounds from thyroid surgery in the neck or laparoscopic surgery in the upper abdomen, excessive bed elevation may subject the wound to pressure, causing discomfort and pain. Conversely, patients who are recovering from surgery for skin tumors or limbs may find a slightly reclined drinking position more suitable. Hence, determining whether to elevate the head of the bed and to what extent should be based on the individual patient’s comfort level.

Early drinking water had a positive effect on several aspects of patient well-being, including stabilizing the patient’s mood, alleviating postoperative tension and anxiety, effectively relieving their thirst, improving the dryness and discomfort in their throats, and reducing postoperative wound pain. Notably, no positive connection was observed between decreased indicator scores and water intake volume, with similar therapeutic impacts observed across different water consumption levels, Notably, there was no linear correlation between the reduction in other scores and the volume of water consumed, and no significant differences were found among groups with varying water intake amounts (*p* > 0.05, Table 3). Even a small amount of warm water given orally was equally effective at achieving the desired therapeutic effect. In essence, different patients consumed varying quantities of water, but the treatment goal remained consistent. Drinking water-stimulated gland secretion effectively alleviated dry throat discomfort, and postoperative patients were satisfied with the early drinking water treatment strategy.

This study employed ultrasound to monitor the movement of the gastric antrum both before and after drinking water. These changes in gastric antrum movement serve as an indicator of gastrointestinal function recovery. Research has demonstrated that a complex bidirectional relationship exists between the brain and the gut. Negative emotions, such as anxiety or fear, can have adverse effects on intestinal function [20, 21]. Patients who are recovering from general anesthesia experience uncertain surgical outcomes, unfamiliar surroundings, pain, thirst, hunger, and other discomfort, which can lead to negative emotions. These emotions directly affect gastrointestinal motility. We observed that most patients had relatively static stomachs before drinking water, with a median gastric antrum MI of 0.00 (0, 0.05). However, after drinking water, patients’ moods stabilized, and they felt happier and more at ease. Drinking water also directly stimulates the gastric wall, promoting gastric peristalsis and gastric secretion through this dual effect. Ultrasound revealed a significant increase in gastric antrum movement, with the median gastric antrum MI after drinking water increased to 1.61 (0,3.66).

This study revealed a positive correlation between antral motility and water intake (Fig. 3; Table 3). The more water patients consumed, the more active their gastrointestinal peristalsis became (*r* = 0.283; *p* < 0.001). The median antral MI of patients who consumed 1–2 ml/kg water was 1.78 (0.45–4.18). The median antral MI of patients who consumed > 2 ml/kg water was 2.35 (0.59–3.77), which was significantly greater than that of patients who consumed < 1 ml/kg water. The linear correlation between water intake and the antral MI, as well as any potential capping effect, is an aspect that has not been addressed in the literature, thus warranting further research.

We can conceptualize a scenario in which after general anesthesia resuscitation, patients are free to drink unlimited amounts of water while in the PACU; however, the amount of water consumed is closely related to the surgical site and sex. Patients who underwent thyroid surgery tended to consume less water at a relatively slower pace, as swallowing could cause discomfort or pain at the surgical site. In contrast, those undergoing superficial or limb surgeries demonstrated a preference for larger quantities and a quicker pace of water intake. Furthermore, male patients consistently expressed greater demands for water than female patients did, leading to greater water intake [22]. The amount of water consumed was also closely related to the patient’s past drinking habits [23]. Patients who habitually consumed more water daily also exhibited greater desire and capacity for post-surgery water consumption.

Drinking large amounts of water does not increase the incidence of nausea and vomiting, but fatal injuries with vomiting aspiration cannot be ruled out. For the sake of clinical safety and rationality, it is still recommended to set a maximum drinking water volume. This not only alleviates the concerns of medical staff but also meets patients’ need for drinking water while achieving the same therapeutic effects. A reasonable drinking water volume is suggested to be ≤ 3 ml/kg of body weight or to maintain the total amount within 300 ml [17].

As with any research, it is important to consider specific constraints. The study’s key limitation is that the data collectors were aware of the study protocol. This non-blinding situation might have caused bias in the data collection process, which could have impacted the objectivity and accuracy of the results. Furthermore, the study encompasses various surgical procedures, and the quantity of water intake is intricately linked to the surgical location and procedure type. Post-anesthesia care unit (PACU) staff members face limitations and are unable to perform pre-drinking evaluations promptly based on the patient’s physiological recovery, potentially leading to significant variations in the time gap for water consumption.

## Conclusion

Patients undergoing non-gastrointestinal surgery can consume a suitable amount of water according to their desire, provided that they meet the criteria for post-general anesthesia recovery. Early water consumption is safe, feasible, and well tolerated. This can address the psychological demands of patients, stabilize their emotions, facilitate gastrointestinal function recovery, and improve overall medical treatment satisfaction.

## Data Availability

Data is provided within the manuscript or supplementary information files.
